# Life without dUTPase

**DOI:** 10.3389/fmicb.2016.01768

**Published:** 2016-11-14

**Authors:** Csaba Kerepesi, Judit E. Szabó, Veronika Papp-Kádár, Orsolya Dobay, Dóra Szabó, Vince Grolmusz, Beáta G. Vértessy

**Affiliations:** ^1^PIT Bioinformatics Group, Institute of Mathematics, Eötvös Loránd UniversityBudapest, Hungary; ^2^Department of Applied Biotechnology and Food Sciences, Budapest University of Technology and EconomicsBudapest, Hungary; ^3^Institute of Enzymology, Research Centre for Natural Sciences, Hungarian Academy of SciencesBudapest, Hungary; ^4^Institute of Medical Microbiology, Semmelweis UniversityBudapest, Hungary; ^5^Uratim Ltd.,Budapest, Hungary

**Keywords:** dUTPase, uracil, Uracil-DNA-glycosylase, U-DNA, UGI, horizontal gene transfer, genome analysis, prokaryotes

## Abstract

Fine-tuned regulation of the cellular nucleotide pools is indispensable for faithful replication of Deoxyribonucleic Acid (DNA). The genetic information is also safeguarded by DNA damage recognition and repair processes. Uracil is one of the most frequently occurring erroneous bases in DNA; it can arise from cytosine deamination or thymine-replacing incorporation. Two enzyme activities are primarily involved in keeping DNA uracil-free: dUTPase (dUTP pyrophosphatase) activity that prevent thymine-replacing incorporation and uracil-DNA glycosylase activity that excise uracil from DNA and initiate uracil-excision repair. Both dUTPase and the most efficient uracil-DNA glycosylase (UNG) is thought to be ubiquitous in free-living organisms. In the present work, we have systematically investigated the genotype of deposited fully sequenced bacterial and Archaeal genomes. We have performed bioinformatic searches in these genomes using the already well described dUTPase and UNG gene sequences. For dUTPases, we have included the trimeric all-beta and the dimeric all-alpha families and also, the bifunctional dCTP (deoxycytidine triphosphate) deaminase-dUTPase sequences. Surprisingly, we have found that in contrast to the generally held opinion, a wide number of bacterial and Archaeal species lack all of the previously described dUTPase gene(s). The *dut*– genotype is present in diverse bacterial phyla indicating that loss of this (or these) gene(s) has occurred multiple times during evolution. We discuss potential survival strategies in lack of dUTPases, such as simultaneous lack or inhibition of UNG and possession of exogenous or alternate metabolic enzymes involved in uracil-DNA metabolism. The potential that genes previously not associated with dUTPase activity may still encode enzymes capable of hydrolyzing dUTP is also discussed. Our data indicate that several unicellular microorganisms may efficiently cope with a *dut*– genotype lacking all of the previously described dUTPase genes, and potentially leading to an unusual uracil-enrichment in their genomic DNA.

## Introduction

The inherent chemical reactivity of DNA and the presence of reactive metabolites and other molecular species within the cell leads to numerous chemical modifications within the DNA even under normal, physiological conditions (Lindahl, 1993; Lu et al., 2001; Bohr et al., 2002; Vertessy and Toth, 2009). Mutations arising from these modifications need to be kept under control, and numerous DNA damage recognition and repair processes evolved to deal with these problems (Meier and Gartner, 2014). However, especially for single cell organisms, eminently for bacteria, increased mutational rates leading to new phenotypes may be even advantageous for the species – appearance of antibiotic resistant strains is a prominent example in this respect (Boshoff et al., 2003; Kana and Mizrahi, 2004). Meanwhile, cells that acquired mutations deleterious for the phenotype will be overgrown by cells with advantageous mutations. In multicellular eukaryotes, such evolutionary changes are more complex since, in these organisms, the viable phenotype is more restricted due to the highly increased interactions within the cellular environment and also with the other cells/organs.

**FIGURE 1:**
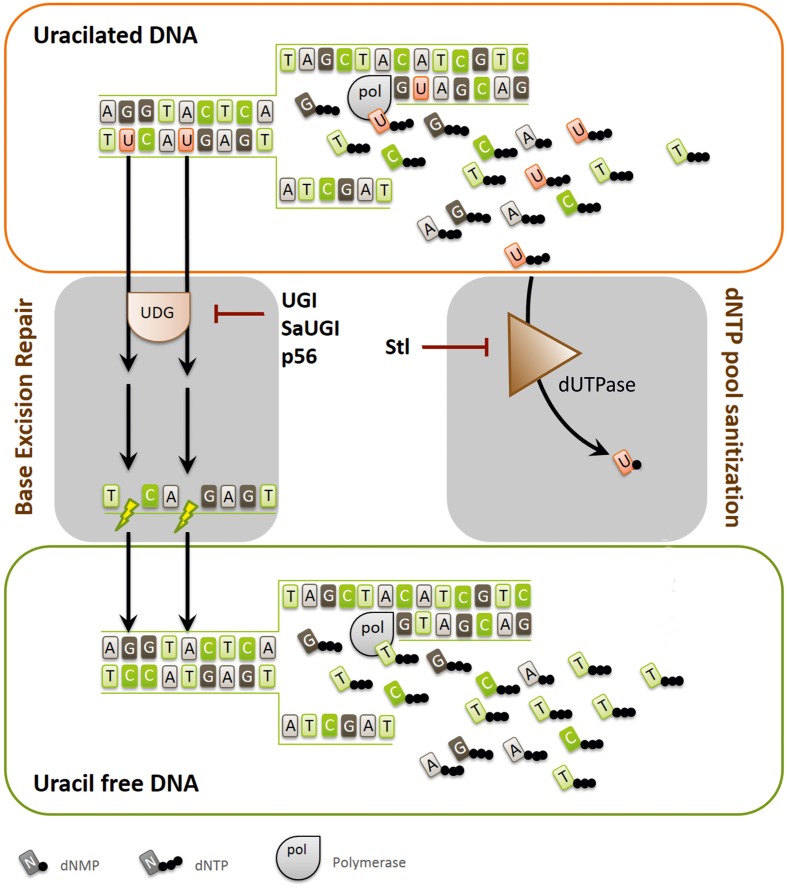
**Pathways and protein factors involved in the metabolism of uracil-substituted DNA.** Uracil may arise in the DNA by cytosine deamination and by dUTP incorporation. The scheme illustrates that dUTPase and UDG are responsible for keeping uracil out of DNA by dNTP pool sanitization or uracil-excision, respectively. Inhibitor proteins against UDG (UGI, SaUGI, and p56) and dUTPase (Stl) are also included on the figure, showing their point of inhibitory attack. The figure also highlights that high uracil content of the DNA can lead to strand breaks, and thus to genomic instability due to the futile cycles of base excision repair.

The DNA repair pathways (Jiricny, 1998), responsible for guarding the DNA-encoded information, are strongly conserved from bacteria to human. The protein factors involved in these processes are usually ubiquitous, although the cognate protein families and isoforms may differ among organisms of different evolutionary branches. For pathways of key significance, it is also frequently observed that multiple protein families with similar functions are present in one organism to safeguard DNA-encoded information (Visnes et al., 2009). In addition to the dedicated DNA damage recognition and repair pathways, sanitization and proper balance of the nucleotide pools are also of high importance (Galperin et al., 2006; Vertessy and Toth, 2009; Nagy et al., 2014). Hence, regulation of nucleotide *de novo* biosynthesis and salvage pathways need to be fine-tuned, and unwanted dNTPs, such as dUTP and dITP have to be removed. There is an intimate cross-talk between enzymes responsible for sanitizing of nucleotide pools and the respective base-excision repair DNA N-glycosylases. These enzymes act hand in hand first to prevent incorporation of the unwanted nucleotide building block containing modified bases into newly synthesizing DNA and second, to excise those moieties that escaped the preventive measure or got produced within the DNA *in situ*. For the uracil moiety, the preventive/excising enzyme activities are presented by the dUTPase and the uracil-DNA glycosylases (UDGs) enzyme families, respectively (Dengg et al., 2006; Castillo-Acosta et al., 2012b; Muha et al., 2012; Pecsi et al., 2012; Szabo et al., 2014). **Figure 1** describes how UDG and dUTPase collaborate to keep DNA uracil-free and also shows the inhibitory protein factors described so far in the literature for either dUTPase or UDG. Note that the UDG enzyme family constitutes several members, among which the catalytically most competent one is abbreviated as UNG.

In a dUTPase knock-out background, viability can be still restored in some cases by simultaneous UNG knock-out (Dengg et al., 2006; Castillo-Acosta et al., 2012a,b), or by inhibiting the UNG enzyme with its specific and highly efficient protein inhibitor, UGI (**Figure 1**). In the double mutant organisms, the uracil content within DNA is highly elevated, however, the cells can survive, most probably since the majority of uracil moieties under these conditions are present as thymine-replacements, i.e., with the same Watson-Crick coding characteristics. Such circumstances have been observed in artificially engineered bacteria (*Escherichia coli*), or similar situations are also found in specific life stages of wild type *Drosophila melanogaster* where dUTPase is down-regulated during development and the *ung* gene is absent from the genome (el-Hajj et al., 1992; Muha et al., 2012). One of the first site-directed mutagenesis methods, introduced by Kunkel is based also on the crosstalk between dUTPase and UNG enzymes, and on the uracilated DNA produced in the artificial *E. coli* strain lacking both dUTPase and UNG activity (Kunkel et al., 1991).

The importance of dUTPase is underlined by its reported ubiquity. However, our recent observations in several *Staphylococcus* strains shed light on circumstances where the dUTPase gene on the bacterial chromosome is present only due to insertion of a phage-encoded gene (in prophage form) (Szabo et al., 2014). Analysis of the genomic information available for numerous Staphylococcal strains (Golding et al., 2012; Chen et al., 2013) also revealed several occasions where strains are viable and infectious in the absence of any dUTPase gene(s) present in the genome (Szabo et al., 2014). Some of these identified bacteria that lack even prophage dUTPases are not just viable and infectious, but are also MRSA (Methicillin Resistant *Staphylococcus aureus*) strains (Golding et al., 2012; Chen et al., 2013), increasing the biomedical significance of our observations related to the genotype of these strains.

This intriguing situation in *S. aureus* prompted us to investigate in details the genotypes of bacteria and Archaea with respect to the existence of genes primarily involved in uracil-DNA metabolism. Besides, the presence of the inhibitory protein factors described so far in the literature for UNG was investigated as well. Results clearly showed that numerous investigated microbes do not possess dUTPase genes, and this genotype can be paired with different patterns of presence/absence of UNG and UNG inhibitor genes. We conclude that the genetic distribution of proteins involved in uracil-DNA metabolism is unexpectedly diverse, and these conditions may have physiological consequences.

## Results

### Several Prokaryotic Genomes Lack dUTPase

For dUTPases, two protein families have been described to date, the all-β trimeric and the all-α dimeric dUTPases (11), hence we used representative sequences of these families in our search (dUTPases from *E. coli* and *Campylobacter jejuni*, respectively). Some Staphylococcal phages also encode a variety of dimeric dUTPase which is less similar to other dimeric dUTPases, hence one such sequence was also inserted in the search. The all-beta trimeric dUTPase family belongs to the dUTPase superfamily which contains also the dCTP deaminase enzymes. dCTP deaminases have the same fold as dUTPases, and some of them were shown to be a bifunctional dCTP deaminase/dUTPase with weak dUTPase activity (Bjornberg et al., 2003; Huffman et al., 2003; Helt et al., 2008). Interestingly, in Archaea it was shown that the protein annotated as dCTP deaminase acts rather as an efficient dUTPase and lacks dCTP deaminase activity (Hogrefe et al., 2002). One such sequence was therefore also included (namely, the sequence annotated as dCTP deaminase from *Pyrococcus furiosus*).

**FIGURE 2:**
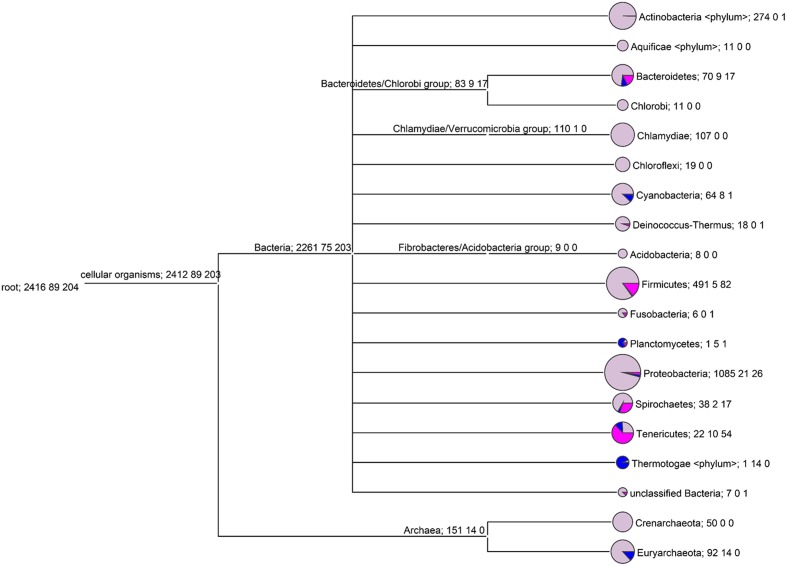
**The distribution of bacterial/Archaeal genomes with and without dUTPase at the phylum level.** Only those phyla are shown that have at least 15 genomes examined. Each node of the tree is labeled by three numbers: the first is the number of genomes with dUTPase under the node (lilac color on the pie graph segment); the second is the number of genomes without both dUTPase and UNG (blue color on the pie graph segment); the third is the number of genomes without dUTPase and with UNG (pink color on the pie graph segment).

In our studies, we investigated those prokaryote genomes that are fully sequenced and deposited in the NCBI Genome database that is, 2261 bacterial and 151 archaeal genomic sequence sets. The result of screening the bacterial and Archaeal genomes for the presence/absence of dUTPase genes is shown in **Figure 2** and in **Supplementary Figure S1**. Interestingly, this systematic approach revealed that the lack of dUTPase genes (dimeric and trimeric dUTPase) is far more frequent than usually thought. Numerous evolutionary taxa showed up where a few or more species do not encode dUTPase protein (note the colored pie graph segments on **Figure 2**: lilac for genomes that possess the dUTPase gene (*dut*+ genotype), blue for genomes lacking both dUTPase and UNG genes (*dut*–*ung*– genotype), and pink for genomes lacking the gene for dUTPase, but possessing the gene for UNG gene (*dut–ung*+ genotype). In fact, most of the phyla contained some species where the dUTPase genes were not found. These instances are widely occurring in Bacteria, and also among Euryarchaeota.

Among the three different genotypes identified in the prokaryote genomes in our study, the *dut*+ genotype is the one that conforms to the usual expectations. Among Bacteria, it is worthwhile to mention that in the Thermotogae phylum, almost all investigated genomes (14 out of 15) also lack the dUTPase genes. The *dut*– genotype is also frequently occurring among Planctomycetes (6 species out 7, i.e., 86%), Tenericutes (64 species out of 86, i.e., over 74%), Spirochaetes (19 species out of 57, i.e., 33 %), Bacteroidetes (26 species out of 96, i.e., 27%), Firmicutes (84 species out of 578, i.e., 14.5 %), and Cyanobacteria (9 species out of 73, i.e., 12%). We have also observed that out of the 106 Euryarchaeota genomes, more than 10% (i.e., 14) lack the dUTPase genes. It is also evident that among the organisms that lack the dUTPase genes, the *dut*–*ung*+ genotype largely outnumber the *dut*–*ung*– genotype in some evolutionary niches (Bacteriodetes, Firmicutes, Spirochaetes, and Tenericutes).

**FIGURE 3:**
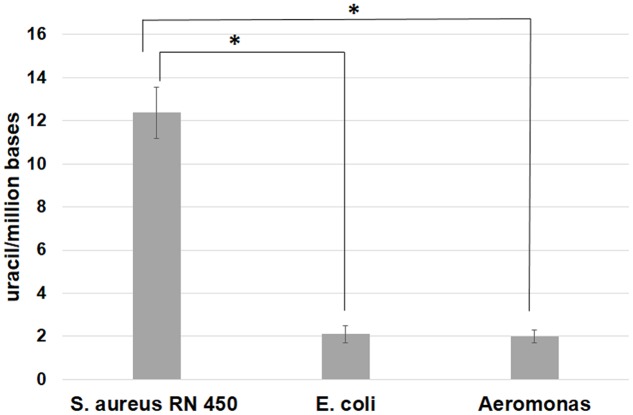
**Genomic uracil-DNA content of *Staphylococcus aureus* RN450, *Escherichia coli* (ATCC 25922), and *Aeromonas hydrophila* (ATCC 7966) strains.** Results were obtained using the uracil-DNA quantification method as described previously (Rona et al., 2016). Significant increase (^∗^) in uracil-DNA content was observed in the data for the *S. aureus* 450 strain as compared to the *E. coli* and *Aeromonas hydrophila* strains (*P* < 0.05). Calculations were based on three independent datasets, representing three different biological samples.

The *dut*– genotype is expected to be associated with an increased uracil content in the DNA genome. To test this expectation, we have analyzed the genomic uracil content in 3 bacteria, namely *S. aureus* (phylum Firmicutes), *E. coli*, and *Aeromonas hydrophila* (phylum Proteobacteria). For the *S. aureus* species, our genome analysis indicated that the strain RN 450 [cured of Staphylococcal phages (Novick, 1967)] does not contain the dUTPase gene [in agreement with (Szabo et al., 2014)], whereas this gene is present in both *E. coli* and *A. hydrophila*. **Figure 3** clearly shows that the experimental results are in agreement with the expectation, namely, the level of uracil in genomic DNA is significantly higher in *S. aureus* samples as compared to either those of *E. coli* or *A. hydrophila*.

## Discussion

### Survival Strategies in the Absence of dUTPase and Possible Physiological Consequences

Our data, despite the usual textbook knowledge, clearly demonstrated that the dUTPase gene is far from being ubiquitous in prokaryotes. It was of immediate further interest to understand how the different organisms may cope with this unexpected situation. We emphasize that our analysis could only involve the dUTPase genes that have been already described in the literature. The proteins encoded in other genes may also possess dUTPase activity, and we will address this possibility also in our discussions under section “Novel protein set for uracil-DNA metabolism.”

#### Simultaneous Lack of UNG Activity

Since the *dut*–*ung*+ genotype is expected to result in genomic instability, it was of interest to investigate if any specific strategy may be employed by the species that are characterized with this unusual feature. The most straightforward survival for the lack of dUTPase activity is the simultaneous absence of UNG activity. Therefore we checked whether the organisms that do not encode a dUTPase possess the *ung* gene or an UNG inhibitor.

##### Lack of the ung gene

For uracil-DNA glycosylase, the sequence of the UNG enzyme from *E. coli* was used in our search, as this subfamily of uracil-DNA glycosylases is associated with the major uracil excising efficiency.

Based on the results the organisms lacking dUTPase gene were further distributed into two groups depending on the simultaneous absence or presence of UNG gene (cf. blue and pink segments on **Figure 2**, for *dut*–*ung*– and *dut*–*ung*+ genotypes, respectively). These two groups are expected to constitute highly different physiological conditions. Simultaneous lack of both dUTPase and UNG (blue segments) possibly results in a viable phenotype with uracil enrichment in the DNA while lack of dUTPase and presence of UNG (pink) is expected to result in genomic instability, and in many cases, cell death.

A more detailed analysis of the evolutionary distribution of species that do not have dUTPase genes is shown in **Supplementary Tables S1** and **S2**. **Table 1** summarizes those evolutionary groups where the occurrence of *dut*– genotypes is detected in >5% of all genomes within the given evolutionary group and also indicates if the UNG gene is present or absent. Note that important pathogens belong also to the groups indicated in **Table 1**.

**Table 1:** Distribution of *dut*– genotypes among bacteria and Archaea.

*dut* – *ung*+	*dut* – *ung* –
Staphylococcaceae	Oscillatoriophycideae
Flavobacteriaceae	Thermoanaerobacterales
Bacillaceae	Oceanospirillales
Enterococcaceae	Mycoplasmataceae
Vibrionaceae	Thermotogaceae
Spirochaetaceae	Methanomicrobia
Mycoplasmataceae	

*Evolutionary branches where the dut– ung+ or dut–ung– genotype occurs in >5% of all genomes within the given evolutionary group.*

##### Presence of an UNG inhibitor

Inhibitory proteins of UNG may modify the physiological scenario, hence we investigated if any of the UNG inhibitory proteins may be encoded in those bacterial and Archaeal genomes that showed up as *dut*–*ung*+ in our analysis (**Supplementary Table S1**).

For UNG, three different proteins have been identified with significant inhibitory effeciency. Two of these (UGI and p56) are encoded by different bacteriophages [phages PBS1/PBS2 and phi29 of *Bacillus subtilis* (Wang and Mosbaugh, 1989) and (Serrano-Heras et al., 2008), respectively]. The UGI function encoded in phages is either required to allow synthesis of uracil-enriched DNA (in the case of phages PBS1/PBS2) or protects against the cleavage of phage genome at uracil positions thereby facilitating viral DNA replication (Serrano-Heras et al., 2008). The third protein with UNG inhibitory activity was recently identified in *S. aureus* (SaUGI) and interestingly, this is the first such case where a UNG inhibitor is encoded in the cellular genome itself (Wang et al., 2014).

We found that none of the phage-related UGI or p56 protein genes could be located on the genomes investigated. The gene for SaUGI, the *S. aureus* UNG inhibitory protein was found in several *S. aureus* strains, and a similar sequence was also found on the *Butyrivibrio proteoclasticus* genome but not elsewhere (**Supplementary Table S1**). Hence, uracil-DNA metabolism basically remains to be governed by the dUTPase and UNG enzymes, with only a very few exceptions, mostly in *S. aureus* strains. The presence of SaUGI protein in *dut*–*ung*+ environment can rescue the potential genomic instability of the bacterium. As UNG inhibitory proteins were earlier detected only in the genome of phages, we wished to check whether this is the case with SaUGI also. Surprisingly we have found that SaUGI is also encoded on a mobile genetic element, namely SCCmec. The same was found and published recently also by the Rice group, in a different context (Mir-Sanchis et al., 2016). The SCCmec element carries the mecA methicillin resistance gene that transforms methicillin susceptible *S. aureus* (MSSA) strains into methicillin resistant (MRSA) strains (Peacock and Paterson, 2015). MRSA strains are a leading cause of health care-associated infections worldwide (Peacock and Paterson, 2015). The fact that such a widespread and important mobile genetic element carries a factor that could rescue the usually lethal *dut*–*ung*+ genotype of its host is fascinating. However, MSSA strains lacking SCCmec are also viable and infectious. Thus, the question emerges why it is advantageous to carry an UNG inhibitor on a mobile genetic element.

Uracil N-Glycosylase inhibitors were previously only identified in *Bacillus subtilis* phages (Wang and Mosbaugh, 1988; Serrano-Heras et al., 2007). In these phages, they either enable synthesis of uracil-enriched DNA or protect against the cleavage of phage genome at uracil positions thereby facilitating viral DNA replication (Cole et al., 2013). Uracilation of viral DNA also plays a role in other host-virus interactions (Chen et al., 2002; Sire et al., 2008). For example, uracilated Human Immunodeficiency Virus (HIV) DNA may be degraded before its integration into the host genome, if the infected host has an active UNG. Thus HIV can only infect cells with low dUTP/dTTP ratio or without an active UNG (Weil et al., 2013). Other viruses, including *S. aureus* phages (Szabo et al., 2014), carry dUTPase, probably to avoid dUTP incorporation in a host with high dUTP/dTTP ratio (cf. Chen et al., 2002; Sire et al., 2008). These findings indicate that although *S. aureus* may be able to maintain its genome integrity in a *dut*– *ung*+ background, mobile genetic elements need dUTPase or SaUGI to avoid uracil-DNA and DNA damage caused by uracil-DNA repair. As heavily uracilated DNA may be degraded by uracil-DNA repair, the presence of these genes on mobile genetic elements suggests that damaging uracil-DNA repair might negatively influences their horizontal gene transfer As horizontal gene transfer has major role in spreading antibiotic resistance and pathogenicity factors in *S. aureus*, the idea that uracil DNA repair could influence this event is intriguing and needs further investigations.

#### Alternate Protein set for Interfering with Uracil-DNA Metabolism

##### Exogenous supply of proteins modifying uracil-DNA metabolism

Interestingly, several bacteriophages carry genes that modify the uracil-DNA metabolism. For example in *S. aureus* strains, all of the prophages encode dUTPase [representatives from either the all-β trimeric or the all-α dimeric dUTPase enzyme families; summarized in Szabo et al. (2014)]. Although the phage dUTPase may be expected to complement the lack of genomic dUTPase, this is not likely to happen *in vivo* due to the life cycle of the phages. *S. aureus* may take advantage of these exogenous dUTPases only in the lysogenic cycle of the phage. However, in this state phage proteins are not expected to be expressed. A study investigating the gene expression pattern of phage proteins upon prophage activation has found that the expression of dUTPase is highly elevated. This indicates that indeed, phage dUTPases are expressed primarily in the lytic cycle. Investigation of the basal expression level of phage dUTPases, and the investigation of dUTP pool and genomic uracil content would be still necessary to decide whether these bacteria may rely on prophage dUTPases.

As mentioned above the UNG inhibitor, UGI was discovered also as a phage protein. The very first finding that led to the identification of UGI was that *Bacillus subtilis* bacteriophages PBS1, and PBS2 possess a genome in which thymine is replaced by uracil (Takahashi and Marmur, 1963). UGI is an early phage protein that prevents degradation by the host UNG and therefore it is indispensable for the maintenance of the uracilated phage genome (Cone et al., 1980; Cole et al., 2013). To date two other bacteriophages have been discovered that possess uracil containing DNA, namely, ΦR1-37 infecting *Y. enterocolitica* (Kiljunen et al., 2005), and phage S6 infecting Staphylococcace (Uchiyama et al., 2014). As none of them encode an already described UNG inhibitor, it is unrevealed to date, how these phages are able to maintain their uracil containing genome. It is possibly that these phages encode a yet undescribed UNG inhibitor.

In summary, several prophages carry genes that encode proteins involved in the uracil metabolism. The products of these genes may modify the scenario predicted based on the genomic sequence of the bacteria.

##### Novel protein set for uracil-DNA metabolism

Another strategy to survive *dut*–*ung*+ genotype may be the use of novel, yet undescribed proteins to supply dUTPase activity or UNG inhibition. Protein evolution is an ongoing process, and specialization of promiscuous nucleotide hydrolyzers to dUTP may have occurred as multiple evolutionary events. For example, the dimeric family of dUTPases, which was discovered much later than the trimeric dUTPase family, was found to belong to the MazG enzyme family that catalyze hydrolytic cleavage of nucleotide phosphates (Moroz et al., 2005). Some MazG-like promiscuous enzymes are able to cleave dUTP among numerous dNTPs, as it was found in *Deinococcus radiodurans* (Goncalves et al., 2011). Although less efficient and less specific, this supplementation of dUTPase enzymatic activity may aid viability. In this respect, it is relevant to point out that in several systems, strong inhibition of dUTPase did not lead to lethality indicating that a residual dUTPase activity might be still enough for survival (Merenyi et al., 2011; Pecsi et al., 2012). Under these circumstances, the genomic DNA may contain a somewhat elevated, but not lethal level of incorporated deoxyuridine moieties. Such an enzyme may have been also the starting point for the evolution of dimeric dUTPases (Goncalves et al., 2011). Low level activity of bifunctional dCTP deaminase-dUTPases may also be sufficient for the survival in the absence of dUTPase. *D. radiodurans* is also known for its high resistance against ionizing radiation (Minton and Daly, 1995). Prokaryotes that are living under extreme circumstances may be important sources for new enzyme activities.

For Thermatoga and Methanomicrobia, data from the literature indicate that the *dut*–*ung*– genotype found in our present work may be compensated for by including genes for a less specific MazG-like dNTPase together with an Archaea-like uracil-DNA glycosylase (Nelson et al., 1999). Lateral gene transfer between Archaea and bacteria has been suggested as the underlying mechanism that led to the appearance of Archaea-like uracil-DNA glycosylase in Thermatoga.

As mentioned earlier, a new UNG inhibitor, SaUGI was also recently described (Wang et al., 2014), and other potential UNG inhibitors in the genome of uracilated phages are still waiting for discovery (Kiljunen et al., 2005; Uchiyama et al., 2014).

## Conclusion

We have shown that the genes for the common dUTPase enzyme families are far from being ubiquitous in prokaryotes. This unexpected genotype is observed in evolutionary well-separated branches suggesting that loss of the *dut* gene(s) might have occurred on multiple independent occasions during evolution. We have also shown the *dut*– genotype is associated with its expected phenotypic increase in genomic uracil content (cf **Figure 3**).

Horizontal gene transfer is of general key importance in spreading virulence elements. In the present study we observe that elements involved in uracil-DNA metabolism are also interestingly found within mobile genetic elements. Parallel spreading of these U-DNA factors with virulence elements may also impact as key regulators of genome integrity and mutagenic rates. The biomedical significance of these findings are especially relevant for microbes of current high therapeutic challenge. Among these, we suggest that depending on the expression pattern of the proteins involved in uracil DNA metabolism, *S. aureus* may have a somewhat uracilated genome and may be genomically instable.

Phages and mobile genetic elements has important role also in lateral gene transfer. For example, the mentioned *S. aureus* S6 phage is a general transducing phage of Staphylococcace. Therefore it would also be even more interesting to further investigate how this phage maintains its genome, and how it may modify the uracil-DNA metabolism of the infected bacteria.

## Materials and Methods

### Analysis of Genomic Data

Here we describe the workflow that has generated the list of bacterial and archaeal genomes without dUTPase and from these genomes those with and without UNG, UGI, SAUGI, and P56. The list, tables and the source of the in-house programs referred below, are available at the website http://pitgroup.org/static/life_wo_dutpase/.

#### Finding Bacterial Genomes that Do Not Contain dUTPase

The source of the bacterial and archaeal genome sequences was downloaded from the NCBI FTP site: ftp://ftp.ncbi.nlm.nih.gov/genomes/Bacteria/all.fna.tar.gz. For sequence search and alignment, the stand-alone UNIX blast program (Altschul et al., 1990) was applied from the site http://www.ncbi.nlm.nih.gov/books/NBK52640/ on our local servers. Next, with the makeblastdb program, databases were generated for the genomic sequences for processing with blast. We filtered out the DNA sequences corresponding to plasmids by applying our in-house scripts GenAllGenomesFileNames.sh and allgenomes_wo-plasmids.pl.

Search for dUTPase sequences, the UNG sequence and the UNG inhibitor UGI-SAUGI-P56 sequences were directed by the run-blast.pl script that calls the program tblastn; the applied fasta files to search for in the database were:

dUTPase-tri-di1-di2-arch.fasta,UNG.fasta, UGI-SAUGI-P56.fasta, all downloadable from http://pitgroup.org/static/life_wo_dutpase/.

The dUTPase fasta file contains one trimeric (*E. coli* dUTPase, UniProt: P06968), two dimeric (*C. jejuni* and *S. aureus* phiEta phage dUTPases, UniProt: O15826 and Q9G011, respectively), as well as and one Archaeal dUTPase-like sequence (the putative dCTP deaminase from *Pyrococcus furiosus*, Uniprot accession number Q8X251). The UNG fasta file contains the NCBI Reference Sequence WP_001262716.1 of Enterobacteriaceae uracil-DNA glycosylase. The fasta file for the UNG inhibitor proteins consists of the sequences corresponding to the UniProt accession numbers P14739, Q936H5, and Q38503.

The evaluation of the tblastn results were performed by the script find-nohits.pl that returned a table of the bacterial/Archaeal genomes without dUTPase genes where no alignments were found with smaller than 0.01 E-value for any of the three dUTPases we search for. The genomes without dUTPase hits were also partitioned into classes (i) according to the containment of UNG genes with better than 0.01 E-value, and (ii) containment of any UNG inhibitors with sequence-similarities from the fasta file UGI-SAUGI-P56.fasta of 0.01 E-value or less. The genomes without dUTPase and with UNG are listed in **Supplementary Table S1**. The memberships in the partitions of (i) and (ii) are denoted in the first two columns of **Supplementary Table S1**. The genomes without both dUTPase and UNG are listed in **Supplementary Table S2**.

The interested reader can easily reproduce the results in each row of **Supplementary Tables S1** and **S2** by using the on-line webserver at NCBI at the site:

http://blast.ncbi.nlm.nih.gov/Blast.cgi?PROGRAM=tblastn&PAGE_TYPE=BlastSearch&LINK_LOC=blasthome by choosing the “*Align two or more sequences*” option, copying the content of the fasta file tri-di1-di2-arch-UNG-UGI-SAUGI-P56.fasta in the first and copying the NC number of the row of the table into the second input field, and setting “Expect threshold” value to 0.01 at the “Algorithm parameters” menu.

#### Generating the Taxonomic Distribution Figure from the Results **Supplementary Tables S1** and **S2**

We have used the MEGAN5 (Huson and Mitra, 2012) metagenomic analysis software in a creative way for generating the evolutionary distribution of the genomes with and without dUTPase and UNG. Certainly, we do not have metagenomes here, but we can exploit a particular capability of the MEGAN5 software as follows. MEGAN5 is capable of comparing the taxonomic distribution of three metagenomes, and it can generate a phylogenetic tree to visualize the distribution. The membership in the three metagenomes can be described by a length-3 0–1 characteristic vector, the *i*th value is 0 if the taxon is not in the metagenome and 1 if it is in the metagenome, for *i*= 1,2,3. Here we substitute these “memberships in metagenomes” with the memberships of sets of genomes with and without dUTPase and UNG as follows: 1,0,0 is substituted if the genome contains dUTPase gene, 0,1,0 is written if the genome does not contain dUTPase but it contain UNG, and 0,0,1 is written if the genome does not contain dUTPase and UNG.

#### Technical Description of the Workflow

First, the file that maps the gi values the Taxonomy IDs was downloaded from the NCBI FTP site: ftp://ftp.ncbi.nlm.nih.gov/pub/taxonomy/gi_taxid_nucl.dmp.gz.From this file, using the non-plasmid bacterial/Archaeal genome-headers, with a script enclosed as Annot-w-TAXID.pl, NC-numbers were mapped to gi and Taxonomy IDs; the resulting file is NC-GI-TAXID-wo-plasmid.csv.

Next, the gen-megan.pl script of ours was applied to get life_wo_di1-di2-tri-arch_dUTPase_E001.megan file that was opened by the MEGAN5 software^1^. The evolutionary tree figures were created by setting the Rank, and in the Tree menu by setting the Show Number of Read Summarized and Show values on log scale options. The leaves, containing only few genomes can be filtered by setting the Tree/Hide Low Support Nodes option in MEGAN5.

### Determination of the Genomic Uracil Content in Selected Bacterial Strains

The *S. aureus* RN450 strain that is cured of prophages (Novick, 1967) and does not contain a dUTPase gene (Szabo et al., 2014) was a kind gift from Prof. Richard P. Novick (New York University, School of Medicine, New York, NY, USA). The *E. coli* (ATCC 25922) and *Aeromonas hydrophila* (ATCC 7966) strains were obtained from ATCC. Bacteria were pre-cultured on blood agar plates overnight at 37°C. To reach the logarithmic phase, cell cultures were inoculated to Brain Heart Infusion media (Sigma), then cultured at 37°C to reach optical density (OD) 0.4–0.5 values. Cells were then harvested and genomic DNA samples were purified with Bacterial genomic ZR Fungal/Bacterial DNA MiniPrep Kit (ZYMO Research, Irvine, CA, USA). The genomic uracil content of the cells were determined as described before (Rona et al., 2016). Briefly, dilution series from a stock solution containing 1 μg of genomic DNA samples from *S. aureus RN450 E. coli* and *Aeromonas hydrophila* cells mixed with 1 μg of carrier salmon sperm DNA were applied on prewetted positively charged nylon membrane (Amersham Hybond- Ny+; GEHealthcare, LittleChalfont, UK) treated as described in Rona et al. (2016). The uracil content of the genomic DNA samples was determined applying the catalytically inactive uracil-DNA glycosylase protein sensor conjugated with Flag tags on the blot membrane. The signal of bound sensor protein was visualized using anti-Flag M2 antibody (Sigma), horseradish peroxidase coupled secondary antibody (Sigma) and enhanced chemiluminescence reagent. Genomic uracil standard samples were prepared using the genomic DNA isolated from log phase CJ236 *E. coli* strain [*dut-,ung*-].

#### Availability of Data And Materials

The source of the bacterial and archaeal genome sequences was downloaded from the NCBI FTP site: ftp://ftp.ncbi.nlm.nih.gov/genomes/Bacteria/all.fna.tar.gz. For sequence search and alignment, the stand-alone UNIX blast program (Altschul et al., 1990) was applied from the site http://www.ncbi.nlm.nih.gov/books/NBK52640/ on our local servers.

Search for dUTPase sequences, the UNG sequence and the UNG inhibitor UGI-SAUGI-P56 sequences were directed by the run-blast.pl script that calls the program tblastn; the applied fasta files to search for in the database were:

dUTPase-tri-di1-di2-arch.fasta,UNG.fasta, UGI-SAUGI-P56.fasta, all downloadable from http://pitgroup.org/static/life_wo_dutpase/.

## Author Contributions

Initiated the study: BV, contributed analysis tools: CK, VP-K, OD, DS, developed software: CK, designed phylogenetic visualization: CK, analyzed results: JS, BV, VG, CK, VP-K, OD, and DS, wrote the paper: BV, VG, JS.

## Conflict of Interest Statement

The authors declare that the research was conducted in the absence of any commercial or financial relationships that could be construed as a potential conflict of interest.

## Footnotes

1 http://ab.inf.uni-tuebingen.de/software/megan5/

## Supplementary Material

FIGURE S1 **The distribution of bacterial/Archaeal genomes with and without dUTPase at the family level.** Only those families are shown that have at least 15 genomes examined. Each node of the tree is labeled by three numbers: the first is the number of genomes with dUTPase under the node (lilac color on the pie graph segment); the second is the number of genomes without both dUTPase and UNG (blue color on the pie graph segment); the third is the number of genomes without dUTPase and with UNG (pink color on the pie graph segment).

TABLE S1 **List of prokaryotic genomes where the *dut* gene is absent and the *ung* gene is present (*dut*–, *ung*+ genotype).** The table provides gives the list of the prokaryotic (bacterial/archaeal) genomes without the dUTPase but with the UNG gene. The second column shows the presence of UNG inhibitors in the genome.

TABLE S2 **List of prokaryotic genomes with the simultaneous lack of the *dut* and *ung* genes (*dut*–*, ung*– genotype).** The table provides gives the list of the prokaryotic (bacterial/archaeal) genomes that lack both the dUTPase and UNG genes.

## Abbreviations

dCTP: deoxycytidine triphosphate
dITP: deoxyinozitol triphosphate
DNA: Deoxyribonucleic Acid
dNTP: deoxynucleoside triphosphate
dNTPase: deoxynucleoside triphosphate hydrolase
dUTP: deoxyuracil triphosphate
dUTPase: dUTP pyrophosphatase
MazG: the name of a nucleotide hydrolase enzyme family
MPD4: name of an UDG enzyme family
MUG: mismatch-specific DNA glycosylase
p56: protein 56, name of an UNG inhibitor protein
RNA: Ribonucleic Acid
SaUGI: Staphylococcus aureus uracil DNA glycosylase inhibitor
SMUG: single-strand-selective monofunctional uracil-DNA glycosylase
Stl: name of a S. aureus pathogenicity island repressor protein
TDG: Thymine DNA glycosylase
UDG: uracil DNA glycosylase
UGI: uracil DNA glycosylase inhibitor
UNG: N-Glycosylase
UNG: uracil N-Glycosylase, the name of the most common uracil DNA glycosylase inhibitor.
