# Supplementary figures and images for: Life without dUTPase
Source: Front Microbiol. 2016 Nov 14;7:1768. doi: 10.3389/fmicb.2016.01768 (PMC5122711; doi:10.3389/fmicb.2016.01768)

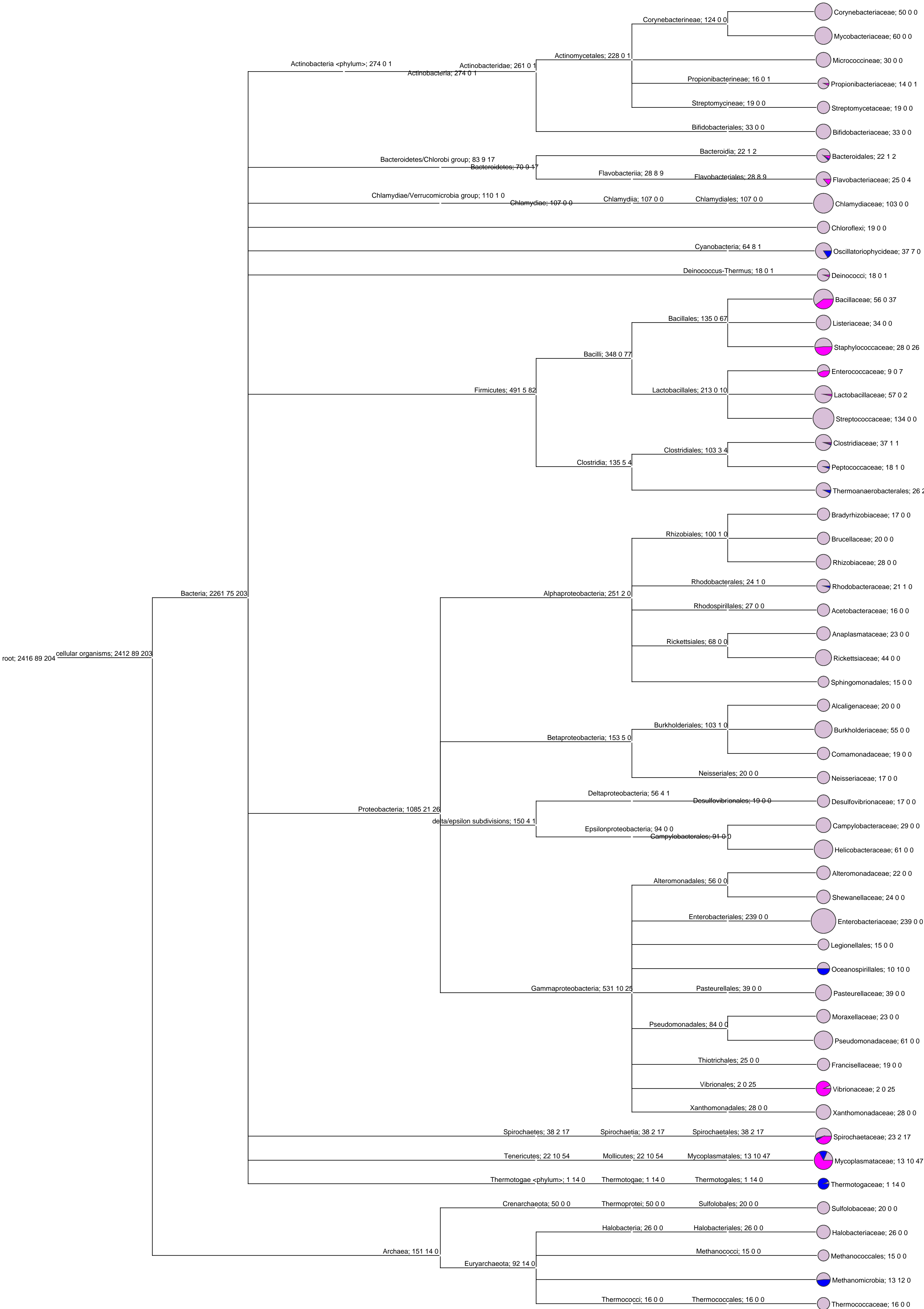

Supplement: FIGURE S1 — The distribution of bacterial/Archaeal genomes with and without dUTPase at the family level. Only those families are shown that have at least 15 genomes examined. Each node of the tree is labeled by three numbers: the first is the number of genomes with dUTPase under the node (lilac color on the pie graph segment); the second is the number of genomes without both dUTPase and UNG (blue color on the pie graph segment); the third is the number of genomes without dUTPase and with UNG (pink color on the pie graph segment). [file Image_1.PDF]
