# Supplementary material for: Life without dUTPase
Source: Front Microbiol. 2016 Nov 14;7:1768. doi: 10.3389/fmicb.2016.01768 (PMC5122711; doi:10.3389/fmicb.2016.01768)
Supplement: TABLE S1 — List of prokaryotic genomes where the dut gene is absent and the ung gene is present (dut–, ung+ genotype). The table provides gives the list of the prokaryotic (bacterial/archaeal) genomes without the dUTPase but with the UNG gene. The second column shows the presence of UNG inhibitors in the genome. [file Table_1.PDF]

UNG (EV=0.01)	UGI or SAUGI or P56 (EV=0.01)	Genome directory name	Acc number	Sequence name	Acc number	Sequence name				
yes	yes	Staphylococcus_epidermidis_ATCC_12228_uid57861	NC_004461	Staphylococcus epidermidis ATCC 12228 chromosome, complete genome						
yes	yes	Halobacillus_halophilus_DSM_2266_uid162033	NC_017668	Halobacillus halophilus DSM 2266, complete genome						
yes	yes	Staphylococcus_haemolyticus_JCSC1435_uid62919	NC_007168	Staphylococcus haemolyticus JCSC1435 chromosome, complete genome						
yes	yes	Butyrivibrio_proteoclasticus_B316_uid51489	NC_014388	Butyrivibrio proteoclasticus B316 chromosome 2, complete genome	NC_014387	Butyrivibrio proteoclasticus B316 chromosome 1, complete genome				
yes	yes	Staphylococcus_aureus_SA40_uid221289	NC_022443	Staphylococcus aureus subsp. aureus SA40, complete genome						
yes	yes	Staphylococcus_epidermidis_RP62A_uid57663	NC_002976	Staphylococcus epidermidis RP62A, complete genome						
yes	yes	Staphylococcus_saprophyticus_ATCC_15305_uid58411	NC_007350	Staphylococcus saprophyticus subsp. saprophyticus ATCC 15305, complete genome						
yes	no	Enterococcus_faecalis_Symbioflor_1_uid183342	NC_019770	Enterococcus faecalis str. Symbioflor 1, complete genome						
yes	no	Spiroplasma_syrphidicola_EA_1_uid205054	NC_021284	Spiroplasma syrphidicola EA-1, complete genome						
yes	no	Mycoplasma_hyorhinis_SK76_uid181997	NC_019552	Mycoplasma hyorhinis SK76 chromosome, complete genome						
yes	no	Spiroplasma_taiwanense_CT_1_uid212975	NC_021846	Spiroplasma taiwanense CT-1, complete genome						
yes	no	Dyadobacter_fermentans_DSM_18053_uid59049	NC_013037	Dyadobacter fermentans DSM 18053 chromosome, complete genome						
yes	no	Borrelia_garinii_NMJW1_uid177081	NC_018747	Borrelia garinii NMJW1 chromosome, complete genome						
yes	no	Staphylococcus_aureus_VC40_uid88071	NC_016912	Staphylococcus aureus subsp. aureus VC40 chromosome, complete genome						
yes	no	Mycoplasma_hyopneumoniae_7448_uid58039	NC_007332	Mycoplasma hyopneumoniae 7448 chromosome, complete genome						
yes	no	Mycoplasma_genitalium_M6320_uid173370	NC_018497	Mycoplasma genitalium M6320 chromosome, complete genome						
yes	no	Vibrio_harveyi_ATCC_BAA_1116_uid58957	NC_009784	Vibrio harveyi ATCC BAA-1116 chromosome II, complete sequence	NC_009783	Vibrio harveyi ATCC BAA-1116 chromosome I, complete sequence				
yes	no	Mycoplasma_hyorhinis_GDL_1_uid87003	NC_016829	Mycoplasma hyorhinis GDL-1 chromosome, complete genome						
yes	no	Borrelia_burgdorferi_B31_uid57581	NC_001318	Borrelia burgdorferi B31 chromosome, complete genome						
yes	no	Melissococcus_plutonius_ATCC_35311_uid66803	NC_015516	Melissococcus plutonius ATCC 35311 chromosome, complete genome						
yes	no	Enterococcus_faecalis_62_uid159663	NC_017312	Enterococcus faecalis 62 chromosome, complete genome	NC_017732	Enterococcus phage EF62phi, complete genome				
yes	no	Vibrio_anguillarum_775_uid68057	NC_015637	Vibrio anguillarum 775 chromosome II, complete sequence	NC_015633	Vibrio anguillarum 775 chromosome I, complete sequence				
yes	no	Borrelia_bissettii_DN127_uid71231	NC_015921	Borrelia bissettii DN127 chromosome, complete genome						
yes	no	Vibrio_fischeri_ES114_uid58163	NC_006840	Vibrio fischeri ES114 chromosome I, complete sequence	NC_006841	Vibrio fischeri ES114 chromosome II, complete sequence				
yes	no	Bacillus_megaterium_QM_B1551_uid15862	NC_014019	Bacillus megaterium QM B1551 chromosome, complete genome						
yes	no	Mycoplasma_pneumoniae_M129_B7_uid185759	NC_020076	Mycoplasma pneumoniae M129-B7, complete genome						
yes	no	Blattabacterium__Blatta_orientalis__Tarazona_uid188115	NC_020195	Blattabacterium sp. (Blatta orientalis) str. Tarazona, complete genome						
yes	no	Mycoplasma_crocodyli_MP145_uid47087	NC_014014	Mycoplasma crocodyli MP145 chromosome, complete genome						
yes	no	Pediococcus_pentosaceus_ATCC_25745_uid57981	NC_008525	Pediococcus pentosaceus ATCC 25745, complete genome						
yes	no	Mycoplasma_bovis_HB0801_uid168665	NC_018077	Mycoplasma bovis HB0801 chromosome, complete genome						
yes	no	Spiroplasma_chrysopicola_DF_1_uid205053	NC_021280	Spiroplasma chrysopicola DF-1, complete genome						
yes	no	Aliivibrio_salmonicida_LFI1238_uid59251	NC_011313	Aliivibrio salmonicida LFI1238 chromosome 2, complete sequence	NC_011312	Aliivibrio salmonicida LFI1238 chromosome chromosome 1, complete sequence				
yes	no	Vibrio_harveyi_ATCC_BAA_1116_uid218471	NC_022270	Vibrio campbellii ATCC BAA-1116 chromosome II, complete sequence	NC_022269	Vibrio campbellii ATCC BAA-1116 chromosome I, complete sequence				
yes	no	Pediococcus_pentosaceus_SL4_uid227215	NC_022780	Pediococcus pentosaceus SL4, complete genome						
yes	no	Vibrio_cholerae_O1_2010EL_1786_uid78933	NC_016445	Vibrio cholerae O1 str. 2010EL-1786 chromosome 1, complete sequence	NC_016446	Vibrio cholerae O1 str. 2010EL-1786 chromosome 2, complete sequence				
yes	no	Borrelia_burgdorferi_N40_uid161241	NC_017418	Borrelia burgdorferi N40 chromosome, complete genome						
yes	no	Bacillus_pumilus_SAFR_032_uid59017	NC_009848	Bacillus pumilus SAFR-032 chromosome, complete genome						
yes	no	Exiguobacterium_AT1b_uid59093	NC_012673	Exiguobacterium sp. AT1b chromosome, complete genome						
yes	no	Mycoplasma_bovis_PG45_uid60859	NC_014760	Mycoplasma bovis PG45 chromosome, complete genome						
yes	no	Vibrio_alginolyticus_NBRC_15630___ATCC_17749_uid199933	NC_022359	Vibrio alginolyticus NBRC 15630 = ATCC 17749 chromosome 2, complete sequence	NC_022349	Vibrio alginolyticus NBRC 15630 = ATCC 17749 chromosome 1, complete sequence				
yes	no	Vibrio_parahaemolyticus_RIMD_2210633_uid57969	NC_004605	Vibrio parahaemolyticus RIMD 2210633 chromosome 2, complete sequence	NC_004603	Vibrio parahaemolyticus RIMD 2210633 chromosome 1, complete sequence				
yes	no	Mycoplasma_genitalium_M2321_uid173373	NC_018495	Mycoplasma genitalium M2321 chromosome, complete genome						
yes	no	Enterococcus_faecalis_OG1RF_uid54927	NC_017316	Enterococcus faecalis OG1RF chromosome, complete genome						
yes	no	Staphylococcus_aureus_6850_uid217772	NC_022222	Staphylococcus aureus subsp. aureus 6850, complete genome						
yes	no	Mycoplasma_pneumoniae_FH_uid162027	NC_017504	Mycoplasma pneumoniae FH chromosome, complete genome						
yes	no	Propionibacterium_freudenreichii_shermanii_CIRM_BIA1_uid49535	NC_014215	Propionibacterium freudenreichii subsp. shermanii CIRM-BIA1 chromosome, complete genome						
yes	no	Borrelia_duttonii_Ly_uid58791	NC_011229	Borrelia duttonii Ly, complete genome						
yes	no	Vibrio_cholerae_M66_2_uid59355	NC_012580	Vibrio cholerae M66-2 chromosome II, complete sequence	NC_012578	Vibrio cholerae M66-2 chromosome I, complete sequence				
yes	no	Haliscomenobacter_hydrossis_DSM_1100_uid66777	NC_015510	Haliscomenobacter hydrossis DSM 1100 chromosome, complete genome						
yes	no	Exiguobacterium_sibiricum_255_15_uid58053	NC_010556	Exiguobacterium sibiricum 255-15 chromosome, complete genome						
yes	no	Blattabacterium__Blaberus_giganteus__uid165873	NC_017924	Blattabacterium sp. (Blaberus giganteus) chromosome, complete genome						
yes	no	Mycoplasma_agalactiae_uid46679	NC_013948	Mycoplasma agalactiae chromosome, complete genome						
yes	no	Mycoplasma_hyopneumoniae_168_L_uid205052	NC_021283	Mycoplasma hyopneumoniae 168-L, complete genome						
yes	no	Mycoplasma_bovis_Hubei_1_uid68691	NC_015725	Mycoplasma bovis Hubei-1 chromosome, complete genome						
yes	no	Ureaplasma_parvum_serovar_3_ATCC_700970_uid57711	NC_002162	Ureaplasma parvum serovar 3 str. ATCC 700970 chromosome, complete genome						
yes	no	Listonella_anguillarum_M3_uid217771	NC_022223	Listonella anguillarum M3 chromosome 1, complete sequence	NC_022224	Listonella anguillarum M3 chromosome 2, complete sequence				
yes	no	Ureaplasma_parvum_serovar_3_ATCC_27815_uid58887	NC_010503	Ureaplasma parvum serovar 3 str. ATCC 27815 chromosome, complete genome						
yes	no	Enterococcus_7L76_uid197170	NC_021023	Enterococcus sp. 7L76 draft genome						
yes	no	Weissella_koreensis_KACC_15510_uid68837	NC_015759	Weissella koreensis KACC 15510 chromosome, complete genome						
yes	no	Emticicia_oligotrophica_DSM_17448_uid177079	NC_018748	Emticicia oligotrophica DSM 17448 chromosome, complete genome						
yes	no	Mycoplasma_hyopneumoniae_168_uid162053	NC_017509	Mycoplasma hyopneumoniae 168 chromosome, complete genome						
yes	no	Ureaplasma_urealyticum_serovar_10_ATCC_33699_uid59011	NC_011374	Ureaplasma urealyticum serovar 10 str. ATCC 33699 chromosome, complete genome						
yes	no	Mycoplasma_genitalium_G37_uid57707	NC_000908	Mycoplasma genitalium G37 chromosome, complete genome						
yes	no	Deinococcus_radiodurans_R1_uid57665	NC_001263	Deinococcus radiodurans R1 chromosome 1, complete sequence	NC_001264	Deinococcus radiodurans R1 chromosome 2, complete sequence				
yes	no	Vibrio_cholerae_MJ_1236_uid59387	NC_012667	Vibrio cholerae MJ-1236 chromosome 2, complete genome	NC_012668	Vibrio cholerae MJ-1236 chromosome 1, complete sequence				
yes	no	Clostridium_acidurici_9a_uid176126	NC_018664	Clostridium acidurici 9a chromosome, complete genome						
yes	no	Blattabacterium__Mastotermes_darwiniensis__MADAR_uid77127	NC_016146	Blattabacterium sp. (Mastotermes darwiniensis) str. MADAR chromosome, complete genome						
yes	no	Borrelia_burgdorferi_ZS7_uid59429	NC_011728	Borrelia burgdorferi ZS7, complete genome						
yes	no	Leadbetterella_byssophila_DSM_17132_uid60161	NC_014655	Leadbetterella byssophila DSM 17132 chromosome, complete genome						
yes	no	Blattabacterium__Periplaneta_americana__BPLAN_uid41287	NC_013418	Blattabacterium sp. (Periplaneta americana) str. BPLAN, complete genome						
yes	no	Mycoplasma_arthritidis_158L3_1_uid58005	NC_011025	Mycoplasma arthritidis 158L3-1 chromosome, complete genome						
yes	no	Vibrio_cholerae_O395_uid58425	NC_009457	Vibrio cholerae O395 chromosome 2, complete sequence	NC_009456	Vibrio cholerae O395 chromosome 1, complete sequence				
yes	no	Runella_slithyformis_DSM_19594_uid68317	NC_015703	Runella slithyformis DSM 19594 chromosome, complete genome						
yes	no	Bacillus_licheniformis_9945A_uid207072	NC_021362	Bacillus licheniformis 9945A, complete genome						
yes	no	Mycoplasma_mobile_163K_uid58077	NC_006908	Mycoplasma mobile 163K, complete genome						
yes	no	Mycoplasma_synoviae_53_uid58061	NC_007294	Mycoplasma synoviae 53, complete genome						
yes	no	Mycoplasma_hominis_ATCC_23114_uid41875	NC_013511	Mycoplasma hominis ATCC 23114 chromosome, complete genome						
yes	no	Candidatus_Nitrospira_defluvii_uid51175	NC_014355	Candidatus Nitrospira defluvii, complete genome						
yes	no	Alistipes_finegoldii_DSM_17242_uid168180	NC_018011	Alistipes finegoldii DSM 17242 chromosome, complete genome						
yes	no	Vibrio_vulnificus_YJ016_uid58007	NC_005139	Vibrio vulnificus YJ016 chromosome I, complete sequence	NC_005140	Vibrio vulnificus YJ016 chromosome II, complete sequence				
yes	no	_Nostoc_azollae__0708_uid49725	NC_014248	'Nostoc azollae' 0708 chromosome, complete genome						
yes	no	Borrelia_afzelii_PKo_uid58653	NT_167352	Borrelia afzelii PKo clone fragment c	NT_167351	Borrelia afzelii PKo clone fragment b	NT_167350	Borrelia afz	NC_008277	Borrelia afzelii PKo, complete genome
yes	no	Borrelia_garinii_BgVir_uid162165	NC_017717	Borrelia garinii BgVir chromosome linear, complete sequence						
yes	no	Bacillus_licheniformis_DSM_13___ATCC_14580_uid58199	NC_006322	Bacillus licheniformis DSM 13 = ATCC 14580 chromosome, complete genome						
yes	no	Eubacterium_eligens_ATCC_27750_uid59171	NC_012778	Eubacterium eligens ATCC 27750 chromosome, complete genome						
yes	no	Enterococcus_faecalis_V583_uid57669	NC_004668	Enterococcus faecalis V583 chromosome, complete genome						
yes	no	Mycoplasma_cynos_C142_uid184824	NC_019949	Mycoplasma cynos C142 complete genome						
yes	no	Bacillus_licheniformis_ATCC_14580_uid58097	NC_006270	Bacillus licheniformis ATCC 14580 chromosome, complete genome						
yes	no	Mycoplasma_pneumoniae_M129_uid57709	NC_000912	Mycoplasma pneumoniae M129 chromosome, complete genome						
yes	no	Erysipelothrix_rhusiopathiae_Fujisawa_uid68021	NC_015601	Erysipelothrix rhusiopathiae str. Fujisawa chromosome, complete genome						
yes	no	Vibrio_vulnificus_MO6_24_O_uid62243	NC_014966	Vibrio vulnificus MO6-24/O chromosome II, complete sequence	NC_014965	Vibrio vulnificus MO6-24/O chromosome I, complete sequence				
yes	no	Mycoplasma_genitalium_M2288_uid173372	NC_018498	Mycoplasma genitalium M2288 chromosome, complete genome						
yes	no	Vibrio_cholerae_IEC224_uid89389	NC_016944	Vibrio cholerae IEC224 chromosome I, complete sequence	NC_016945	Vibrio cholerae IEC224 chromosome II, complete sequence				
yes	no	Mycoplasma_hyorhinis_HUB_1_uid51695	NC_014448	Mycoplasma hyorhinis HUB-1 chromosome, complete genome						
yes	no	Vibrio_splendidus_LGP32_uid59353	NC_011744	Vibrio splendidus LGP32 chromosome 2, complete sequence	NC_011753	Vibrio splendidus LGP32 chromosome 1, complete sequence				
yes	no	Vibrio_Ex25_uid41601	NC_013456	Vibrio sp. Ex25 chromosome 1, complete sequence	NC_013457	Vibrio sp. Ex25 chromosome 2, complete genome				
yes	no	Borrelia_crocidurae_Achema_uid162335	NC_017808	Borrelia crocidurae str. Achema chromosome, complete genome						
yes	no	Vibrio_cholerae_O395_uid159869	NC_012583	Vibrio cholerae O395 chromosome chromosome II, complete sequence	NC_012582	Vibrio cholerae O395 chromosome chromosome I, complete sequence				
yes	no	Borrelia_afzelii_PKo_uid159867	NC_017238	Borrelia afzelii PKo chromosome, complete genome						
yes	no	candidate_division_SR1_bacterium_RAAC1_SR1_1_uid230714	NC_023002	Candidate division SR1 bacterium RAAC1_SR1_1, complete genome						
yes	no	Alistipes_shahii_WAL_8301_uid197175	NC_021030	Alistipes shahii WAL 8301 draft genome						
yes	no	Mycoplasma_hyopneumoniae_7422_uid212968	NC_021831	Mycoplasma hyopneumoniae 7422, complete genome						
yes	no	Mycoplasma_hyorhinis_DBS_1050_uid228933	NC_022807	Mycoplasma hyorhinis DBS 1050, complete genome						
yes	no	Mycoplasma_pneumoniae_309_uid85495	NC_016807	Mycoplasma pneumoniae 309, complete genome						
yes	no	Enterococcus_faecalis_D32_uid171261	NC_018221	Enterococcus faecalis D32 chromosome, complete genome						
yes	no	Isosphaera_pallida_ATCC_43644_uid62207	NC_014962	Isosphaera pallida ATCC 43644 chromosome, complete genome						
yes	no	Mycoplasma_genitalium_M6282_uid173371	NC_018496	Mycoplasma genitalium M6282 chromosome, complete genome						
yes	no	Vibrio_cholerae_O1_biovar_El_Tor_N16961_uid57623	NC_002505	Vibrio cholerae O1 biovar El Tor str. N16961 chromosome I, complete sequence	NC_002506	Vibrio cholerae O1 biovar El Tor str. N16961 chromosome II, complete sequence				
yes	no	Vibrio_parahaemolyticus_BB22OP_uid184822	NC_019955	Vibrio parahaemolyticus BB22OP chromosome 1, complete sequence	NC_019971	Vibrio parahaemolyticus BB22OP chromosome 2, complete sequence				
yes	no	Mycoplasma_conjunctivae_uid59325	NC_012806	Mycoplasma conjunctivae HRC/581 chromosome, complete genome						
yes	no	Borrelia_garinii_PBi_uid58125	NC_006156	Borrelia garinii PBi chromosome linear, complete sequence						

yes	no	Borrelia_miyamotoi_LB_2001_uid215233	NC_022079	Borrelia miyamotoi LB-2001, complete genome		
yes	no	Vibrio_EJY3_uid83161	NC_016613	Vibrio sp. EJY3 chromosome 1, complete sequence	NC_016614	Vibrio sp. EJY3 chromosome 2, complete sequence
yes	no	Exiguobacterium_MH3_uid227425	NC_022794	Exiguobacterium sp. MH3, complete genome		
yes	no	Bacillus_megaterium_DSM319_uid48371	NC_014103	Bacillus megaterium DSM 319 chromosome, complete genome		
yes	no	Mycoplasma_agalactiae_PG2_uid61619	NC_009497	Mycoplasma agalactiae PG2 chromosome, complete genome		
yes	no	Mycoplasma_pulmonis_UAB_CTIP_uid61569	NC_002771	Mycoplasma pulmonis UAB CTIP, complete genome		
yes	no	Vibrio_furnissii_NCTC_11218_uid82347	NC_016602	Vibrio furnissii NCTC 11218 chromosome 1, complete sequence	NC_016628	Vibrio furnissii NCTC 11218 chromosome 2, complete sequence
yes	no	Borrelia_burgdorferi_JD1_uid161197	NC_017403	Borrelia burgdorferi JD1 chromosome, complete genome		
yes	no	Borrelia_afzelii_HLJ01_uid177930	NC_018887	Borrelia afzelii HLJ01 chromosome, complete genome		
yes	no	Blattabacterium__Blattella_germanica__Bge_uid41533	NC_013454	Blattabacterium sp. (Blattella germanica) str. Bge, complete genome		
yes	no	Geobacillus_Y4_1MC1_uid55779	NC_014650	Geobacillus sp. Y4.1MC1 chromosome, complete genome		
yes	no	Vibrio_vulnificus_CMCP6_uid62909	NC_004459	Vibrio vulnificus CMCP6 chromosome I, complete sequence	NC_004460	Vibrio vulnificus CMCP6 chromosome II, complete sequence
yes	no	Vibrio_fischeri_MJ11_uid58907	NC_011184	Vibrio fischeri MJ11 chromosome I, complete sequence	NC_011186	Vibrio fischeri MJ11 chromosome II, complete sequence
yes	no	Borrelia_recurrentis_A1_uid58793	NC_011244	Borrelia recurrentis A1, complete genome		
yes	no	Mycoplasma_hyopneumoniae_J_uid58059	NC_007295	Mycoplasma hyopneumoniae J chromosome, complete genome		
yes	no	Borrelia_burgdorferi_CA382_uid214794	NC_022048	Borrelia burgdorferi CA382, complete genome		
yes	no	Geobacillus_thermoglucosidasius_C56_YS93_uid48129	NC_015660	Geobacillus thermoglucosidasius C56-YS93 chromosome, complete genome		
yes	no	Desulfurivibrio_alkaliphilus_AHT2_uid49487	NC_014216	Desulfurivibrio alkaliphilus AHT2 chromosome, complete genome		
yes	no	Vibrio_nigripulchritudo_SnF1_uid222819	NC_022543	Vibrio nigripulchritudo str. SFn1 chromosome, complete genome	NC_022528	Vibrio nigripulchritudo str. SFn1 chromosome, complete genome
yes	no	Vibrio_cholerae_LMA3984_4_uid159541	NC_017269	Vibrio cholerae LMA3984-4 chromosome II, complete sequence	NC_017270	Vibrio cholerae LMA3984-4 chromosome chromosome I, complete sequence
yes	no	Photobacterium_profundum_SS9_uid62923	NC_006370	Photobacterium profundum SS9 chromosome 1, complete genome	NC_006371	Photobacterium profundum SS9 chromosome 2, complete sequence
yes	no	Melissococcus_plutonius_DAT561_uid89371	NC_016938	Melissococcus plutonius DAT561 chromosome 1, complete genome		
yes	no	Spirosoma_linguale_DSM_74_uid43413	NC_013730	Spirosoma linguale DSM 74 chromosome, complete genome		
yes	no	Erysipelothrix_rhusiopathiae_SY1027_uid206518	NC_021354	Erysipelothrix rhusiopathiae SY1027, complete genome		
yes	no	Spiroplasma_diminutum_CUAS_1_uid212976	NC_021833	Spiroplasma diminutum CUAS-1, complete genome		
yes	no	Mycoplasma_hyorhinis_MCLD_uid162087	NC_017519	Mycoplasma hyorhinis MCLD chromosome, complete genome		
yes	no	Mycoplasma_hyopneumoniae_232_uid58205	NC_006360	Mycoplasma hyopneumoniae 232 chromosome, complete genome		
yes	no	Leptotrichia_buccalis_C_1013_b_uid59211	NC_013192	Leptotrichia buccalis C-1013-b chromosome, complete genome		
