# Supplementary material for: Life without dUTPase
Source: Front Microbiol. 2016 Nov 14;7:1768. doi: 10.3389/fmicb.2016.01768 (PMC5122711; doi:10.3389/fmicb.2016.01768)
Supplement: TABLE S2 — List of prokaryotic genomes with the simultaneous lack of the dut and ung genes (dut–, ung– genotype). The table provides gives the list of the prokaryotic (bacterial/archaeal) genomes that lack both the dUTPase and UNG genes. [file Table_2.PDF]

Genome directory name	Acc number	Sequence name	Acc number	Sequence name
Candidatus_Carsonella_ruddii_DC_uid213383	NC_021894	Candidatus Carsonella ruddii DC, complete genome		
Mahella_australiensis_50_1_BON_uid66917	NC_015520	Mahella australiensis 50-1 BON chromosome, complete genome		
Candidatus_Portiera_aleyrodidarum_BT_QVLC_uid176374	NC_018676	Candidatus Portiera aleyrodidarum BT-QVLC chromosome, complete genome		
Candidatus_Portiera_aleyrodidarum_BT_B_uid176373	NC_018677	Candidatus Portiera aleyrodidarum BT-B-HRs chromosome, complete genome		
Candidatus_Portiera_aleyrodidarum_BT_QVLC_uid175570	NC_018618	Candidatus Portiera aleyrodidarum BT-QVLC chromosome, complete genome		
Candidatus_Portiera_aleyrodidarum_BT_B_uid173859	NC_018507	Candidatus Portiera aleyrodidarum BT-B chromosome, complete genome		
Thermotoga_neapolitana_DSM_4359_uid59065	NC_011978	Thermotoga neapolitana DSM 4359 chromosome, complete genome		
Mycoplasma_haemofelis_Langford_1_uid62461	NC_014970	Mycoplasma haemofelis str. Langford 1, complete genome		
Candidatus_Sulcia_muelleri_DMIN_uid47075	NC_014004	Candidatus Sulcia muelleri DMIN chromosome, complete genome		
Dactylococcopsis_salina_PCC_8305_uid183341	NC_019780	Dactylococcopsis salina PCC 8305 chromosome, complete genome		
Thermotoga_petrophila_RKU_1_uid58655	NC_009486	Thermotoga petrophila RKU-1 chromosome, complete genome		
Candidatus_Carsonella_ruddii_uid58773	NC_008512	Candidatus Carsonella ruddii PV, complete genome		
Candidatus_Tremblaya_phenacola_PAVE_uid209173	NC_021555	Candidatus Tremblaya phenacola PAVE, complete genome		
Pseudanabaena_PCC_7367_uid183004	NC_019701	Pseudanabaena sp. PCC 7367 chromosome, complete genome		
Thermotoga_elfii_NBRC_107921_uid227422	NC_022792	Thermotoga elfii NBRC 107921 DNA, complete genome		
Thermotoga_RQ2_uid58935	NC_010483	Thermotoga sp. RQ2 chromosome, complete genome		
Archaeoglobus_sulfaticallidus_PM70_1_uid201033	NC_021169	Archaeoglobus sulfaticallidus PM70-1, complete genome		
Mesotoga_prima_MesG1_Ag_4_2_uid52599	NC_017934	Mesotoga prima MesG1.Ag.4.2 chromosome, complete genome		
Candidatus_Carsonella_ruddii_HT_isolate_Thao2000_uid172735	NC_018417	Candidatus Carsonella ruddii HT isolate Thao2000 chromosome, complete genome		
Methanosarcina_mazei_Go1_uid57893	NC_003901	Methanosarcina mazei Go1 chromosome, complete genome		
Methanococcoides_burtonii_DSM_6242_uid58023	NC_007955	Methanococcoides burtonii DSM 6242, complete genome		
Candidatus_Hodgkinia_cicadicola_Dsem_uid59311	NC_012960	Candidatus Hodgkinia cicadicola Dsem chromosome, complete genome		
Methanolobus_psychrophilus_R15_uid177925	NC_018876	Methanolobus psychrophilus R15 chromosome, complete genome		
Planctomyces_brasiliensis_DSM_5305_uid60583	NC_015174	Planctomyces brasiliensis DSM 5305 chromosome, complete genome		
Candidatus_Tremblaya_princeps_PCVAL_uid159519	NC_017293	Candidatus Tremblaya princeps PCVAL chromosome, complete genome		
Mycoplasma_wenyonii_Massachusetts_uid170731	NC_018149	Mycoplasma wenyonii str. Massachusetts chromosome, complete genome		
Methanocella_arvoryzae_MRE50_uid61623	NC_009464	Methanocella arvoryzae MRE50 chromosome, complete genome		
Methanosarcina_acetivorans_C2A_uid57879	NC_003552	Methanosarcina acetivorans C2A chromosome, complete genome		
Candidatus_Carsonella_ruddii_HC_isolate_Thao2000_uid172734	NC_018416	Candidatus Carsonella ruddii HC isolate Thao2000 chromosome, complete genome		
Methanomethylovorans_hollandica_DSM_15978_uid184864	NC_019977	Methanomethylovorans hollandica DSM 15978, complete genome		
Chroococcidiopsis_thermalis_PCC_7203_uid183002	NC_019695	Chroococcidiopsis thermalis PCC 7203 chromosome, complete genome		
Thermotoga_thermarum_DSM_5069_uid68449	NC_015707	Thermotoga thermarum DSM 5069 chromosome, complete genome		
Candidatus_Carsonella_ruddii_CE_isolate_Thao2000_uid172732	NC_018414	Candidatus Carsonella ruddii CE isolate Thao2000 chromosome, complete genome		
Thermotoga_maritima_MSB8_uid57723	NC_000853	Thermotoga maritima MSB8 chromosome, complete genome		
Blattabacterium__Nauphoeta_cinerea__uid222815	NC_022550	Blattabacterium sp. (Nauphoeta cinerea), complete genome		
Candidatus_Uzinura_diaspidicola_ASNER_uid186740	NC_020135	Candidatus Uzinura diaspidicola str. ASNER, complete genome		
Candidatus_Zinderia_insecticola_CARI_uid52459	NC_014497	Candidatus Zinderia insecticola CARI chromosome, complete genome		
Mycoplasma_ovis_Michigan_uid232247	NC_023062	Mycoplasma ovis str. Michigan, complete genome		
Fervidobacterium_pennivorans_DSM_9078_uid78143	NC_017095	Fervidobacterium pennivorans DSM 9078 chromosome, complete genome		
Planctomyces_limnophilus_DSM_3776_uid48643	NC_014148	Planctomyces limnophilus DSM 3776 chromosome, complete genome		
Singulisphaera_acidiphila_DSM_18658_uid81777	NC_019892	Singulisphaera acidiphila DSM 18658 chromosome, complete genome		
uncultured_Termite_group_1_bacterium_phylotype_Rs_D17_uid59059	NC_020419	Uncultured Termite group 1 bacterium phylotype Rs-D17 DNA, complete genome	NS_000191	Uncultured Termite group 1 bacterium phylotype Rs-D17, complete genome
Archaeoglobus_fulgidus_DSM_4304_uid57717	NC_000917	Archaeoglobus fulgidus DSM 4304 chromosome, complete genome		
Mycoplasma_haemofelis_Ohio2_uid162029	NC_017520	Mycoplasma haemofelis Ohio2 chromosome, complete genome		
Clostridium_botulinum_BKT015925_uid66203	NC_015425	Clostridium botulinum BKT015925 chromosome, complete genome		
Akkermansia_muciniphila_ATCC_BAA_835_uid58985	NC_010655	Akkermansia muciniphila ATCC BAA-835 chromosome, complete genome		
Thermosipho_melanesiensis_BI429_uid58683	NC_009616	Thermosipho melanesiensis BI429 chromosome, complete genome		
Mycoplasma_suis_Illinois_uid61897	NC_015155	Mycoplasma suis str. Illinois chromosome, complete genome		
Thermotoga_naphthophila_RKU_10_uid42777	NC_013642	Thermotoga naphthophila RKU-10, complete genome		
Desulfotomaculum_kuznetsovii_DSM_6115_uid67357	NC_015573	Desulfotomaculum kuznetsovii DSM 6115 chromosome, complete genome		
Candidatus_Sulcia_muelleri_SMDSEM_uid59393	NC_013123	Candidatus Sulcia muelleri SMDSEM, complete genome		
Thermotoga_maritima_MSB8_uid202924	NC_021214	Thermotoga maritima MSB8, complete genome		
Mycoplasma_suis_KI3806_uid63665	NC_015153	Mycoplasma suis KI3806, complete genome		
Spirochaeta_caldaria_DSM_7334_uid68753	NC_015732	Spirochaeta caldaria DSM 7334 chromosome, complete genome		
Candidatus_Sulcia_muelleri_GWSS_uid58943	NC_010118	Candidatus Sulcia muelleri GWSS, complete genome		
Thermotoga_lettingae_TMO_uid58419	NC_009828	Thermotoga lettingae TMO chromosome, complete genome		
Microcystis_aeruginosa_NIES_843_uid59101	NC_010296	Microcystis aeruginosa NIES-843 chromosome, complete genome		
Candidatus_Sulcia_muelleri_Sulcia_ALF_uid214083	NC_021916	Candidatus Sulcia muelleri str. Sulcia-ALF, complete genome		
Thermosipho_africanus_TCF52B_uid59095	NC_011653	Thermosipho africanus TCF52B chromosome, complete genome		
Thermotoga_hypogea_NBRC_106472_uid227423	NC_022795	Thermotoga hypogea NBRC 106472 DNA, complete genome		
Candidatus_Sulcia_muelleri_CARI_uid52535	NC_014499	Candidatus Sulcia muelleri CARI chromosome, complete genome		
Candidatus_Nasuia_deltocephalinicola_NAS_ALF_uid214084	NC_021919	Candidatus Nasuia deltocephalinicola str. NAS-ALF, complete genome		
Kosmotoga_olearia_TBF_19_5_1_uid59205	NC_012785	Kosmotoga olearia TBF 19.5.1, complete genome		
Candidatus_Portiera_aleyrodidarum_TV_uid195460	NC_020831	Candidatus Portiera aleyrodidarum TV, complete genome		
Methanocella_conradii_HZ254_uid157911	NC_017034	Methanocella conradii HZ254 chromosome, complete genome		
Thermacetogenium_phaeum_DSM_12270_uid177811	NC_018870	Thermacetogenium phaeum DSM 12270 chromosome, complete genome		
Desulfobacca_acetoxidans_DSM_11109_uid65785	NC_015388	Desulfobacca acetoxidans DSM 11109 chromosome, complete genome		
Cyanobacterium_aponinum_PCC_10605_uid183340	NC_019776	Cyanobacterium aponinum PCC 10605 chromosome, complete genome		
Fervidobacterium_nodosum_Rt17_B1_uid58625	NC_009718	Fervidobacterium nodosum Rt17-B1 chromosome, complete genome		
Desulfobacula_toluolica_Tol2_uid175777	NC_018645	Desulfobacula toluolica Tol2, complete genome		
Spirochaeta_thermophila_DSM_6578_uid162041	NC_017583	Spirochaeta thermophila DSM 6578 chromosome, complete genome		
Methanohalophilus_mahii_DSM_5219_uid47313	NC_014002	Methanohalophilus mahii DSM 5219 chromosome, complete genome		
Methanosarcina_barkeri_Fusaro_uid57715	NC_007355	Methanosarcina barkeri str. Fusaro, complete genome		

Synechococcus_PCC_6312_uid182934	NC_019680	Synechococcus sp. PCC 6312 chromosome, complete genome
Petrotoga_mobilis_SJ95_uid58747	NC_010003	Petrotoga mobilis SJ95 chromosome, complete genome
Candidatus_Mycoplasma_haemolamae_Purdue_uid171259	NC_018219	Candidatus Mycoplasma haemolamae str. Purdue chromosome, complete genome
Candidatus_Mycoplasma_haemominutum__Birmingham_1__uid197195	NC_021007	Candidatus Mycoplasma haemominutum 'Birmingham 1' noncontiguous finished genome
Pirellula_staleyi_DSM_6068_uid43209	NC_013720	Pirellula staleyi DSM 6068 chromosome, complete genome
Thermotoga_maritima_MSB8_uid179902	NC_023151	Thermotoga maritima MSB8, complete genome
Mycoplasma_haemocanis_Illinois_uid82367	NC_016638	Mycoplasma haemocanis str. Illinois chromosome, complete genome
Oscillibacter_valericigenes_Sjm18_20_uid73895	NC_016048	Oscillibacter valericigenes Sjm18-20, complete genome
Syntrophobacter_fumaroxidans_MPOB_uid58177	NC_008554	Syntrophobacter fumaroxidans MPOB chromosome, complete genome
Bacteroides_uniformis_uid13130	NC_006373	Bacteroides uniformis mobilizable transposon NBU1, complete sequence
Cyanobacterium_stanieri_PCC_7202_uid183337	NC_019778	Cyanobacterium stanieri PCC 7202 chromosome, complete genome
Cyanothece_PCC_8801_uid59027	NC_011726	Cyanothece sp. PCC 8801 chromosome, complete genome
Rhodobacter_sphaeroides_ATCC_17025_uid58451	NC_009428	Rhodobacter sphaeroides ATCC 17025 chromosome, complete genome
Methanohalobium_evestigatum_Z_7303_uid49857	NC_014253	Methanohalobium evestigatum Z-7303 chromosome, complete genome
Methanosarcina_mazei_Tuc01_uid190185	NC_020389	Methanosarcina mazei Tuc01, complete genome
Candidatus_Carsonella_ruddii_CS_isolate_Thao2000_uid172733	NC_018415	Candidatus Carsonella ruddii CS isolate Thao2000 chromosome, complete genome
Methanocella_paludicola_SANAE_uid42887	NC_013665	Methanocella paludicola SANAE chromosome, complete genome
Prevotella_oral_taxon_299_F0039_uid45899	NC_022124	Prevotella sp. oral taxon 299 str. F0039, complete genome
Mycoplasma_parvum_Indiana_uid223379	NC_022575	Mycoplasma parvum str. Indiana, complete genome
Desulfobacterium_autotrophicum_HRM2_uid59061	NC_012108	Desulfobacterium autotrophicum HRM2 chromosome, complete genome
Blattabacterium__Cryptocercus_punctulatus__Cpu_uid81083	NC_016621	Blattabacterium sp. (Cryptocercus punctulatus) str. Cpu chromosome, complete genome
Candidatus_Tremblaya_princeps_PCIT_uid68741	NC_015736	Candidatus Tremblaya princeps PCIT chromosome, complete genome
Rhodopirellula_baltica_SH_1_uid61589	NC_005027	Rhodopirellula baltica SH 1 chromosome, complete genome
Candidatus_Carsonella_ruddii_PC_isolate_NHV_uid172736	NC_018418	Candidatus Carsonella ruddii PC isolate NHV chromosome, complete genome
